# Minimally Invasive Versus Open CRS/HIPEC for Peritoneal Surface Malignancies: Postoperative Recovery and Long-Term Outcomes—A Systematic Review and Meta-Analysis

**DOI:** 10.3390/cancers18203310

**Published:** 2026-10-14

**Authors:** Matteo Aulicino, Vincenzo Tondolo, Carlo Abatini, Giorgio D’Annibale, Lorenzo Barberis, Maria Saiz, Claudio Fiorillo, Davide De Sio, Giuseppe Quero, Sergio Alfieri, Fabio Pacelli, Francesco Santullo

**Affiliations:** 1 Pancreatic Surgery Unit, Fondazione Policlinico Agostino Gemelli IRCCS of Rome, 00168 Rome, Italy; matteo.aulicino@guest.policlinicogemelli.it (M.A.); claudio.fiorillo@policlinicogemelli.it (C.F.); davide.desio@policlinicogemelli.it (D.D.S.); giuseppe.quero@policlinicogemelli.it (G.Q.); sergio.alfieri@policlinicogemelli.it (S.A.); 2 Dipartimento di Medicina e Chirurgia Traslazionale, Università Cattolica del Sacro Cuore di Roma, 00168 Rome, Italy; carlo.abatini@policlinicogemelli.it (C.A.); fabio.pacelli@policlinicogemelli.it (F.P.); francesco.santullo@policlinicogemelli.it (F.S.); 3 General Surgery, Department of Medical and Surgical Sciences, Fondazione Policlinico Universitario Agostino Gemelli IRCCS, 00168 Rome, Italy; 4 Surgical Unit of Peritoneum and Retroperitoneum, Fondazione Policlinico Universitario Agostino Gemelli IRCCS, Largo Agostino Gemelli 8, 00168 Rome, Italy; giorgio.dannibale@guest.policlinicogemelli.it (G.D.); lorenzo.barberis@guest.policlinicogemelli.it (L.B.); 5 MEMOTEF Department, Sapienza University of Rome, 00161 Rome, Italy; maria.saiz@uniroma1.it

**Keywords:** minimally invasive surgery, cytoreductive surgery, hyperthermic intraperitoneal chemotherapy, peritoneal surface malignancies, systematic review, meta-analysis

## Abstract

*Background*: Minimally invasive surgery (MIS) is increasingly implemented for cytoreductive surgery (CRS) combined with hyperthermic intraperitoneal chemotherapy (HIPEC) for peritoneal surface malignancies (PSM). This systematic review and meta-analysis compared minimally invasive CRS/HIPEC (M-CRS/HIPEC) and open CRS/HIPEC (O-CRS/HIPEC) in terms of perioperative, postoperative, and long-term oncological outcomes. *Methods*: A systematic review and meta-analysis were conducted according to PRISMA guidelines. PubMed, Embase, Scopus, Web of Science, and the Cochrane Library were searched for comparative studies of M-CRS/HIPEC versus O-CRS/HIPEC in adult patients. Risk of bias was assessed using the Newcastle–Ottawa Scale and RoB 2.0. Pooled relative risks (RR) for dichotomous outcomes and standardized mean differences (SMD) for continuous outcomes were calculated with 95% confidence intervals (CI). Sensitivity analyses were performed to assess the potential influence of disease burden and primary tumor heterogeneity. *Results*: Twenty-one studies (1 randomized controlled trial and 20 retrospective cohorts) including 1184 patients (452 M-CRS/HIPEC and 732 O-CRS/HIPEC) were analyzed. Operative time did not differ between groups (SMD = 0.195; *p* = 0.667). Most cytoreductive procedures were comparable, although small bowel resections were less frequent with M-CRS/HIPEC (RR = 0.537; *p* = 0.018). The minimally invasive approach achieved higher CC0 rates (RR = 1.053; *p* = 0.045), with lower blood loss (SMD = −1.838; *p* < 0.001) and fewer transfusions (RR = 0.588; *p* = 0.002). Postoperative recovery favored M-CRS/HIPEC, with earlier return of bowel function and resumption of oral intake, shorter hospital stay, fewer readmissions, and earlier chemotherapy resumption (all *p* < 0.05). Overall and major morbidity were reduced, while postoperative mortality and 1-, 3-, and 5-year disease-free and overall survival did not differ significantly between groups. In the disease burden-restricted sensitivity analysis, differences in small bowel resection, CC0 rate, and 90-day readmission were no longer statistically significant, whereas the main perioperative and recovery benefits persisted. No significant differences in DFS or OS were identified in the additional sensitivity analyses at the evaluable time points. *Conclusions*: This meta-analysis supports a selective, expertise-driven use of M-CRS/HIPEC, offering perioperative and recovery advantages without significant differences in cytoreductive adequacy or long-term outcomes in appropriately selected patients.

## Simple Summary

Cytoreductive surgery combined with heated chemotherapy delivered inside the abdomen is an established treatment for selected patients whose cancer has spread to the abdominal lining. Traditionally, this procedure is performed through a conventional laparotomy, but minimally invasive techniques are increasingly used in selected patients. We combined the results of 21 studies including 1184 patients to evaluate whether minimally invasive surgery offers advantages over open surgery. The minimally invasive approach was associated with improved postoperative recovery and lower postoperative complication rates, while no statistically significant differences in long-term survival outcomes were detected between the two approaches. These findings support minimally invasive surgery as an option for selected patients treated in specialized centers, rather than as a replacement for open surgery in patients with extensive or complex disease.

## 1. Introduction

Cytoreductive surgery (CRS) combined with hyperthermic intraperitoneal chemotherapy (HIPEC) has significantly improved oncological outcomes in selected patients with peritoneal surface malignancies (PSM) [1,2,3,4]. Complete resection of macroscopic disease, together with locoregional treatment of residual microscopic tumor burden, has translated into meaningful gains in overall survival (OS) and disease-free survival (DFS), enabling a potentially curative strategy [5,6,7]. However, these procedures are technically demanding and inherently associated with considerable morbidity and mortality, comparable to those reported for other high-risk open oncologic operations [8].

Over the past decades, advances in minimally invasive surgery (MIS), including laparoscopic and robotic techniques, have reshaped the management of several abdominal malignancies, offering perioperative advantages without compromising oncological efficacy [9,10,11,12].

Building on these advances, increasing interest has emerged in extending MIS approaches to CRS and HIPEC. Early reports, mainly involving patients with low peritoneal cancer index (PCI) and favorable tumor biology, have demonstrated the feasibility and safety of minimally invasive CRS/HIPEC [13,14]. Although the available literature largely consists of small, retrospective series, the growing number of published studies reflects expanding adoption of this approach and underscores the need for a more robust evaluation of its role [15,16,17,18].

Given the availability of newly published evidence, this systematic review and meta-analysis aimed to compare minimally invasive and open CRS/HIPEC in terms of feasibility, surgical complexity, perioperative outcomes, and long-term oncological results, thereby better defining the role of MIS in contemporary surgical oncology.

## 2. Materials and Methods

### 2.1. Data Sources, Search Strategy, and Selection Criteria

This systematic review was prospectively registered in the International Prospective Register of Systematic Reviews (PROSPERO), registration number: CRD420261308879, and the full protocol is accessible through the PROSPERO database. A literature search was conducted in September 2025 in accordance with the Preferred Reporting Items for Systematic Reviews and Meta-Analyses (PRISMA) guidelines [19]. A systematic search of PubMed, Embase, Scopus, Web of Science, and the Cochrane Library was performed to identify studies comparing outcomes of minimally invasive cytoreductive surgery (M-CRS) plus HIPEC with those of open cytoreductive surgery (O-CRS) plus HIPEC. Studies that used EPIC instead of HIPEC were also included.

The search terms “cytoreductive surgery,” “HIPEC,” “laparoscopic,” “robotic,” and “minimally invasive” were adapted for each database. The complete search strings for each database are provided in Supplementary Material S1.

Eligible studies included retrospective studies, cohort studies, prospective studies, and randomized controlled trials (RCTs) published in English and involving adult patients (≥18 years). Exclusion criteria were articles not written in English, case reports and case series, studies without full-text availability, systematic reviews and meta-analyses, narrative reviews, editorials, and conference abstracts.

### 2.2. Data Extraction

All retrieved articles were uploaded to the Rayyan platform (https://www.rayyan.ai/). Duplicate records were removed semi-automatically. Each article was independently screened for inclusion or exclusion by two authors through abstract and full-text review. Any disagreement was resolved by consensus among the authors. Eligible studies were selected for final analysis according to the predefined inclusion and exclusion criteria.

The following data were extracted from the included studies and tabulated using Microsoft Excel: first author, year of publication, study design, type of minimally invasive surgical approach, total number of patients, number of patients undergoing M-CRS, number of patients undergoing O-CRS, type of intraperitoneal chemotherapy administered (HIPEC/EPIC), PCI value and primary tumor for the M-CRS and O-CRS groups, type of surgical procedures performed (peritoneal and visceral resections), operative time, completeness of cytoreduction score (CC), blood loss, blood transfusion, time to bowel function recovery, time to oral intake resumption, length of hospital stay, 90-day readmission, time to resumption of systemic chemotherapy, postoperative complications according to Clavien Dindo classification (CD) [20], intraoperative complications, mortality, disease-free survival (DFS), and overall survival (OS).

### 2.3. Risk of Bias Assessment

The quality of non-randomized retrospective cohort studies was independently evaluated by two authors using the Newcastle–Ottawa Scale (NOS). The scoring system ranged from 0 to 9 and was based on the following domains: S1: representativeness of the exposed cohort; S2: selection of the non-exposed cohort; S3: ascertainment of exposure; S4: demonstration that the outcome of interest was not present at the start of the study; C: comparability; O1: assessment of outcome; O2: adequacy of follow-up duration for outcomes to occur; O3: adequacy of follow-up [21].

Randomized controlled trials (RCTs) were evaluated using the RoB 2.0 (Risk of Bias 2.0) tool. RoB 2.0 is a standardized instrument developed by the Cochrane group to assess the risk of bias in randomized studies across five key domains: the randomization process, deviations from intended interventions, missing outcome data, outcome measurement, and selection of the reported results [22].

### 2.4. Outcomes of Interest

This study aimed to compare patients undergoing cytoreductive surgery plus HIPEC via a minimally invasive approach with those treated using an open approach in terms of:

- Type of surgical procedures performed, including visceral and peritoneal resections;
- Perioperative outcomes, including operative time, completeness of cytoreduction, intraoperative blood loss, and perioperative transfusion rates;
- Postoperative recovery, including time to bowel function recovery, time to resumption of oral intake, length of postoperative hospital stay, 90-day readmission, and time to return to systemic chemotherapy;
- Morbidity, including intraoperative complications, overall postoperative complications, major postoperative complications (Clavien-Dindo ≥ III), and postoperative mortality;
- Long-term outcomes, including disease-free survival (DFS) and overall survival (OS).

A first, disease burden-restricted sensitivity analysis was performed to assess the potential impact of differences in peritoneal disease burden and patient selection between the two surgical approaches. The analysis was restricted to retrospective matched studies in which the M-CRS and O-CRS cohorts had comparable PCI values. Within this restricted set of studies, all outcomes for which sufficient data were available for quantitative synthesis were re-evaluated, including surgical procedures, perioperative outcomes, postoperative recovery, morbidity, mortality, and long-term oncological outcomes. A second sensitivity analysis was conducted to assess the potential impact of primary tumor case-mix on long-term oncological outcomes using two complementary approaches:

- *Tumor-specific sensitivity analysis*, in which DFS and OS were separately evaluated in studies exclusively including gastrointestinal or gynecological malignancies, with studies reporting mixed primary tumor populations excluded;
- *Tumor case-mix-restricted sensitivity analysis*, in which DFS and OS were re-evaluated after restricting the analysis to studies showing a comparable distribution of primary tumor types between the M-CRS and O-CRS cohorts, defined by a maximum absolute standardized difference (Max |SMD|) < 0.100 across primary tumor categories or as both groups consisting exclusively of the same primary tumor type.

### 2.5. Certainty of Evidence Assessment

The certainty of evidence for each outcome was assessed using the Grading of Recommendations Assessment, Development and Evaluation (GRADE) framework. Evidence derived from observational studies was initially rated as low certainty and subsequently downgraded or upgraded according to predefined domains, including risk of bias, inconsistency, indirectness, imprecision, and publication bias. The overall certainty of evidence was categorized as high, moderate, low, or very low.

### 2.6. Statistical Analysis

The meta-analysis was performed following the methodological recommendations outlined in the PRISMA (Preferred Reporting Items for Systematic Reviews and Meta-Analyses) guidelines [19].

All analyses were conducted in R version 4.5.1 using the *metafor* package [23]. The choice between fixed-effect and random-effects models was informed by the assessment of between-study heterogeneity. Depending on the presence of between-study heterogeneity, either fixed-effect or random-effects models were fitted. Heterogeneity was evaluated using Cochran’s Q test [24], which assesses whether the dispersion of observed effect sizes exceeds that expected from sampling error alone, and with the *I*^2^ statistic, which estimates (in percent) how much of the total variability in the observed effect sizes can be attributed to heterogeneity among the true effects. While Cochran’s Q was used to assess statistical evidence of heterogeneity, *I*^2^ was used to quantify the magnitude of inconsistency across studies. Accordingly, model selection was based on the overall assessment of between-study heterogeneity rather than on Cochran’s Q test alone. For outcomes showing substantial between-study heterogeneity, a DerSimonian–Laird random-effects model was applied [25], incorporating an estimate of the between-study variance (τ^2^) into the analysis. The random-effects approach was used to account for residual between-study heterogeneity that could not be adequately explained; however, it was not considered a substitute for investigating potential sources of heterogeneity. In the absence of relevant between-study heterogeneity, a fixed-effect model was estimated using the Mantel–Haenszel method [26].

Publication bias was assessed through visual inspection of funnel plots and by applying Egger’s regression test. Comparisons between minimally invasive cytoreductive surgery (M-CRS) and open cytoreductive surgery (O-CRS) were performed using the relative risk (RR) for dichotomous outcomes and the standardized mean difference (SMD) [27] for continuous outcomes, when the primary interest was differences in central tendency between groups.

For long-term oncological endpoints (OS and DFS), survival proportions were pooled as relative risks (RRs) at prespecified time points of 1, 3, and 5 years. Hazard ratios (HRs), generally preferred for time-to-event outcomes, were inconsistently reported across the included studies and could not be reliably reconstructed from the published survival curves because sufficiently detailed information on censoring and numbers at risk was not consistently available. Therefore, fixed-time RRs were used as landmark estimates of survival differences between groups. Point estimates of RR and SMD were considered statistically significant when the *p*-value from the Z test was ≤0.05.

## 3. Results

Using the search strategies described above, 2533 articles were identified (PubMed 285, Embase 786, Scopus 914, Web of Science 465, Cochrane Library 83). After the removal of 1159 duplicates, 1374 records were screened. A total of 1256 articles were excluded after title and abstract screening.

**Figure 1:**
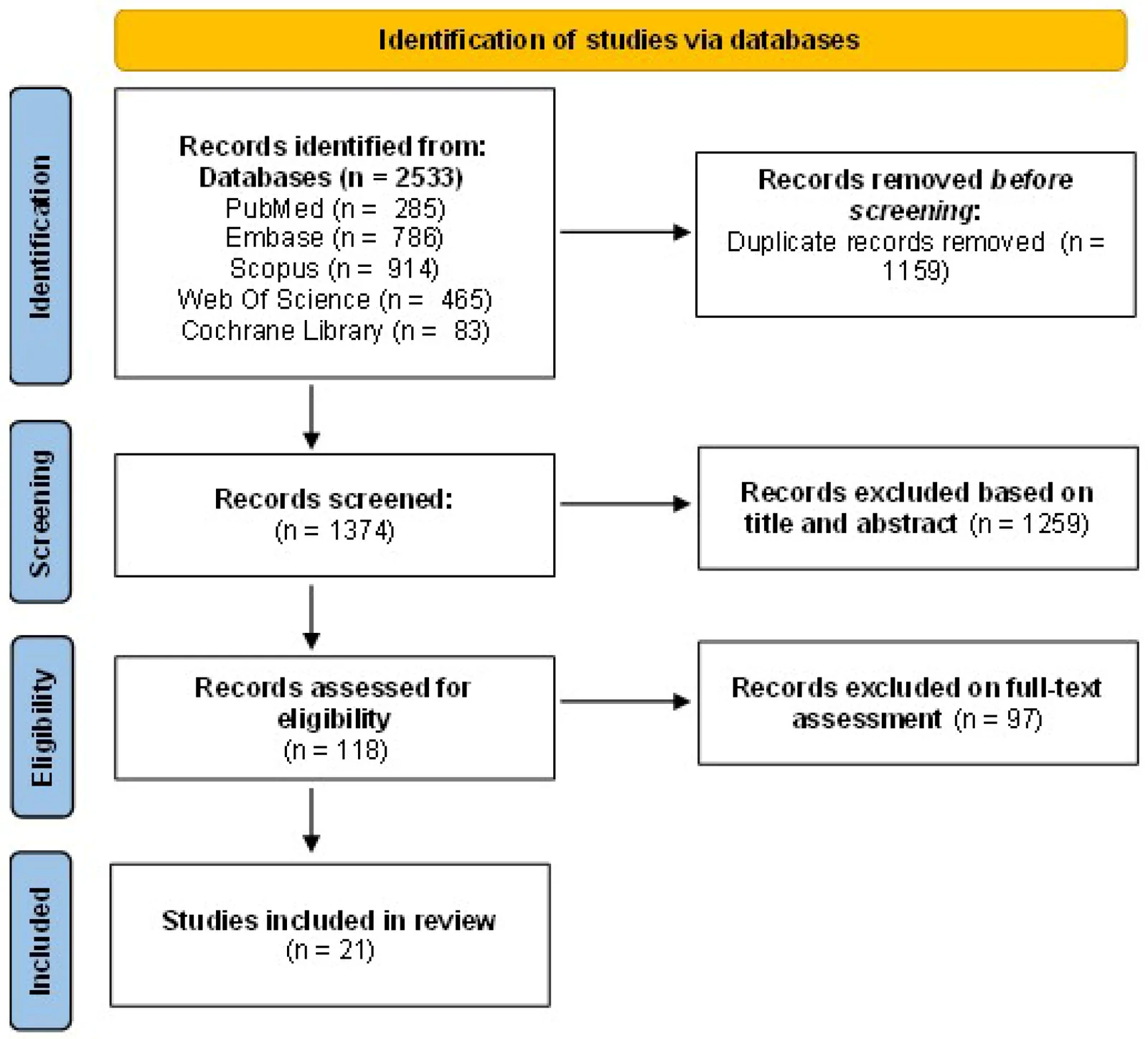
The Preferred Reporting Items for Systematic Review and Meta-Analysis (PRISMA) flowchart.

Of the 118 remaining articles, 97 were excluded following full-text review according to the predefined exclusion criteria. Ultimately, 21 studies were included in this meta-analysis. The PRISMA flow diagram and selection criteria are presented in Figure 1.

A total of 20 retrospective non-randomized cohort studies [18,28,29,30,31,32,33,34,35,36,37,38,39,40,41,42,43,44,45,46] and 1 randomized controlled trial (RCT) [47] were included. All non-randomized studies achieved a Newcastle–Ottawa Scale score of ≥6. According to the RoB 2 tool, the RCT was judged to have an overall risk of bias classified as “some concerns,” driven by a single domain rated as “some concerns,” while all remaining domains were assessed as low risk. Overall, all studies were considered to be of good methodological quality, and the detailed results of the risk-of-bias assessment are provided in Supplementary Material S2.

Table 1 summarizes the main characteristics of the 21 [18,28,29,30,31,32,33,34,35,36,37,38,39,40,41,42,43,44,45,46,47] included studies and their patient populations, comprising a total of 1184 patients, of whom 452 (38.2%) underwent minimally invasive cytoreductive surgery plus HIPEC, while 732 (61.8%) underwent the open approach. Regarding primary tumor type, 10 studies exclusively included gastrointestinal malignancies (studies no. 1, 6, 8, 9, 12, 15, 16, 18, 19, and 20), 4 exclusively included gynecological malignancies (studies no. 2, 5, 11, and 17), while the remaining 7 included mixed primary tumor populations. Eight studies were matched and showed comparable PCI values between the M-CRS and O-CRS groups (studies no. 1, 4, 6, 8, 9, 13, 16, and 17). The maximum absolute standardized difference (Max |SMD|) across primary tumor categories was calculated to assess the comparability of primary tumor case-mix between the two surgical approaches. Overall, 10 of the 21 studies met the predefined criterion for adequately balanced primary tumor case-mix, defined as a Max |SMD| < 0.10 or as both groups consisting exclusively of the same primary tumor type (studies no. 3, 5, 8, 11, 12, 15, 17, 18, 19, and 20).

### 3.1. Evaluation of Surgical Procedures

**Figure 2:**
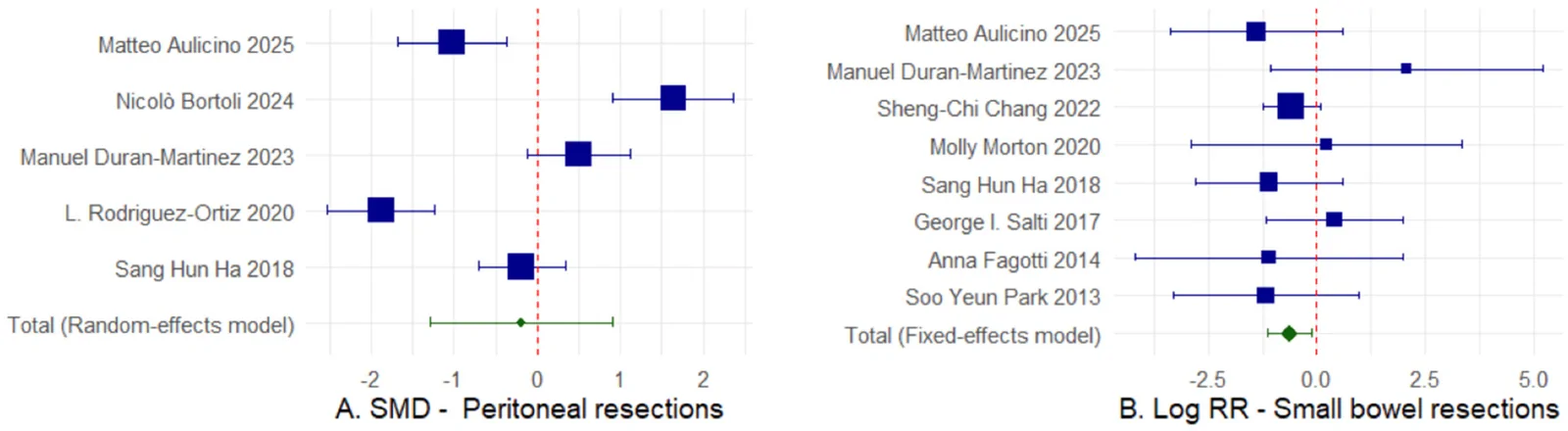
Forest plots comparing surgical procedures performed during CRS between the M-CRS and O-CRS groups: (**A**) Peritoneal resections; (**B**) Small bowel resections. The red dashed line indicates no effect (null value = 0).

Peritoneal resections were reported in 5 studies and showed no statistically significant difference between the M-CRS and O-CRS groups (SMD −0.199; 95% CI −1.300 to 0.901; *p* = 0.722). Heterogeneity was high (*I*^2^ = 93.48%; *p* < 0.001), indicating substantial variability among the included studies, while no evidence of publication bias was detected (*p* = 0.617) (Figure 2A).

Small bowel resections were analyzed in 8 studies and occurred significantly less frequently in the M-CRS group than in the O-CRS group (RR 0.537; 95% CI 0.321–0.900; *p* = 0.018). No heterogeneity was detected (*I*^2^ = 0%; *p* = 0.545), and no evidence of publication bias was identified (*p* = 0.670) (Figure 2B).

No significant differences were observed between groups regarding colonic resections (10 studies; RR 0.956; 95% CI 0.774–1.181; *p* = 0.678), pelvic peritonectomy (4 studies; RR 1.018; 95% CI 0.842–1.230; *p* = 0.857), diaphragmatic peritonectomy (4 studies; RR 0.732; 95% CI 0.393–1.364; *p* = 0.326), hysterectomy/annexectomy (7 studies; RR 0.741; 95% CI 0.391–1.405; *p* = 0.358), gastric resection (4 studies; RR 0.304; 95% CI 0.014–6.446; *p* = 0.445), hepatic resection (5 studies; RR 1; 95% CI 0.445–2.248; *p* = 1.000), splenectomy (3 studies; RR 1.395; 95% CI 0.268–7.260; *p* = 0.693), and stoma creation (6 studies; RR 0.666; 95% CI 0.386–1.149; *p* = 0.693).

### 3.2. Evaluation of Perioperative Outcomes

Operative time was reported in 18 studies. The analysis showed no statistically significant difference between the two groups (SMD 0.195; 95% CI −0.692 to 1.082; *p* = 0.667). Heterogeneity was extremely high (*I*^2^ = 97.36%; *p* < 0.001), indicating substantial methodological or clinical variability among the included studies, whereas no evidence of publication bias was detected (*p* = 0.558).

Completeness of cytoreduction (CC0), reported in 13 studies, was achieved more frequently in the M-CRS group (RR 1.053; 95% CI 1.001–1.107; *p* = 0.045). Heterogeneity was negligible (*I*^2^ = 0%; *p* = 0.962), with no evidence of publication bias (*p* = 0.778).

Intraoperative blood loss, assessed in 14 studies, was significantly lower with M-CRS (SMD −1.838; 95% CI −2.501 to −1.175; *p* < 0.001). Marked heterogeneity was observed (*I*^2^ = 91.03%; *p* < 0.001), whereas Egger’s test did not indicate publication bias (*p* = 0.144).

Perioperative blood transfusions were reported in 9 studies and occurred less frequently in the M-CRS group (RR 0.588; 95% CI 0.417–0.829; *p* = 0.002). No heterogeneity was detected (*I*^2^ = 0%; *p* = 0.811), and publication bias was not observed (*p* = 0.764).

**Figure 3:**
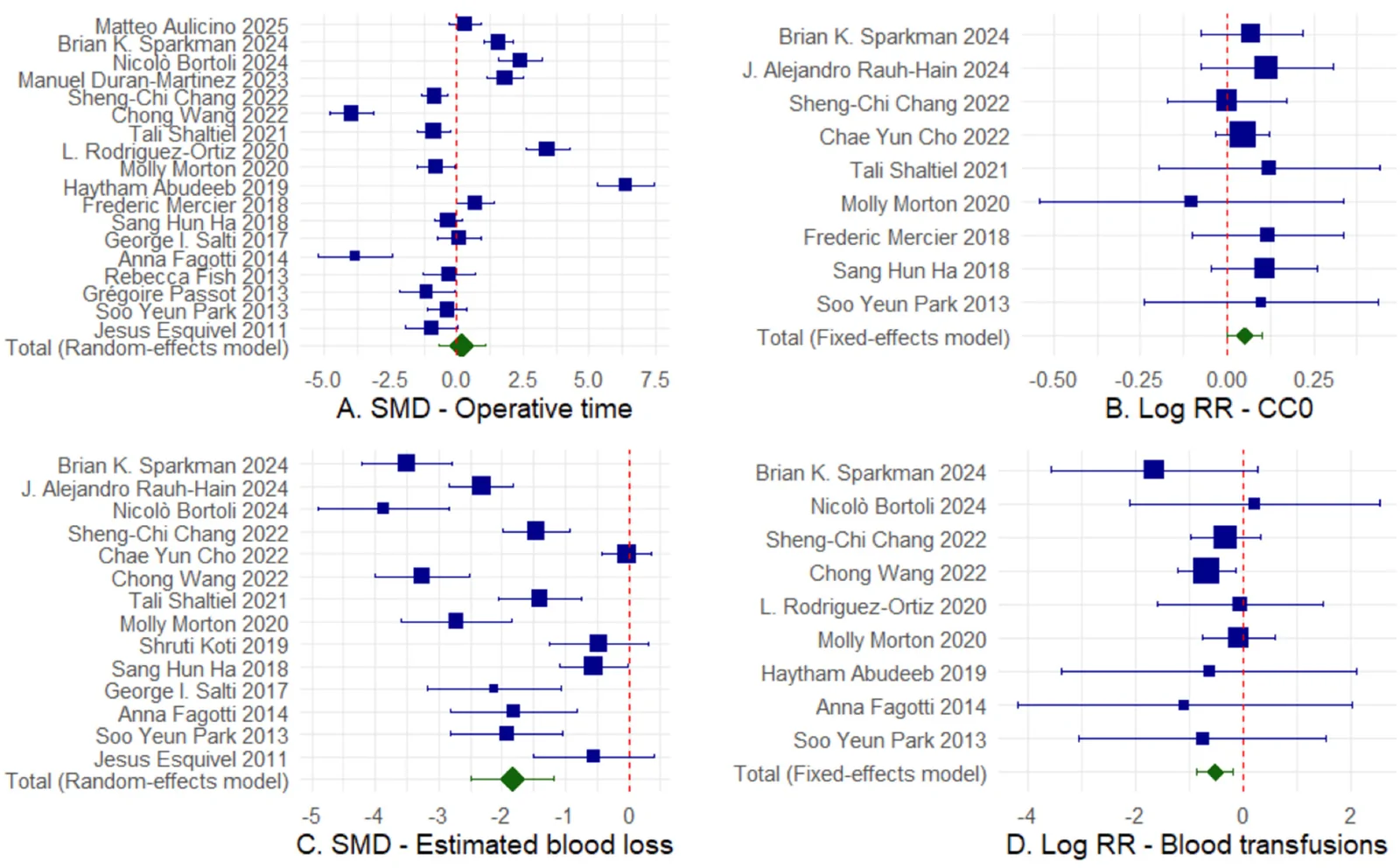
Forest plots comparing perioperative outcomes between the M-CRS and O-CRS groups: (**A**) Operative time; (**B**) CC-0 resection rate; (**C**) Estimated blood loss; (**D**) Perioperative blood transfusions. The red dashed line indicates no effect (null value = 0).

Forest plots of perioperative outcomes comparing the M-CRS and O-CRS groups are presented in Figure 3.

### 3.3. Evaluation of Postoperative Recovery

Return of bowel function was assessed in 5 studies and occurred significantly earlier in the M-CRS group (SMD −0.740; 95% CI −1.128 to −0.351; *p* < 0.001). Moderate heterogeneity was observed (*I*^2^ = 46.94%; *p* = 0.043), with no evidence of publication bias (*p* = 0.087).

Time to oral intake was reported in 3 studies and was significantly shorter in the M-CRS group (SMD −1.242; 95% CI −1.660 to −0.824; *p* < 0.001). No heterogeneity was detected (*I*^2^ = 0.00%; *p* = 0.422), and no evidence of publication bias was observed (*p* = 0.745).

Length of hospital stay was described in 20 studies and was significantly shorter in the M-CRS group (SMD −1.867; 95% CI −2.532 to −1.201; *p* < 0.001). Heterogeneity was high (*I*^2^ = 91.20%; *p* < 0.001), and no evidence of publication bias was observed (*p* = 0.118).

Ninety-day readmission was reported in 9 studies and occurred less frequently in the M-CRS group (RR 0.482; 95% CI 0.249–0.935; *p* = 0.031). No heterogeneity was detected (*I*^2^ = 0.00%; *p* = 0.917), and no evidence of publication bias was found (*p* = 0.742).

Time to resumption of systemic chemotherapy was reported in 5 studies and was significantly shorter in the M-CRS group (SMD −2.479; 95% CI −3.801 to −1.156; *p* < 0.001). High heterogeneity was observed (*I*^2^ = 92.15%; *p* < 0.001), with no evidence of publication bias (*p* = 0.069).

**Figure 4:**
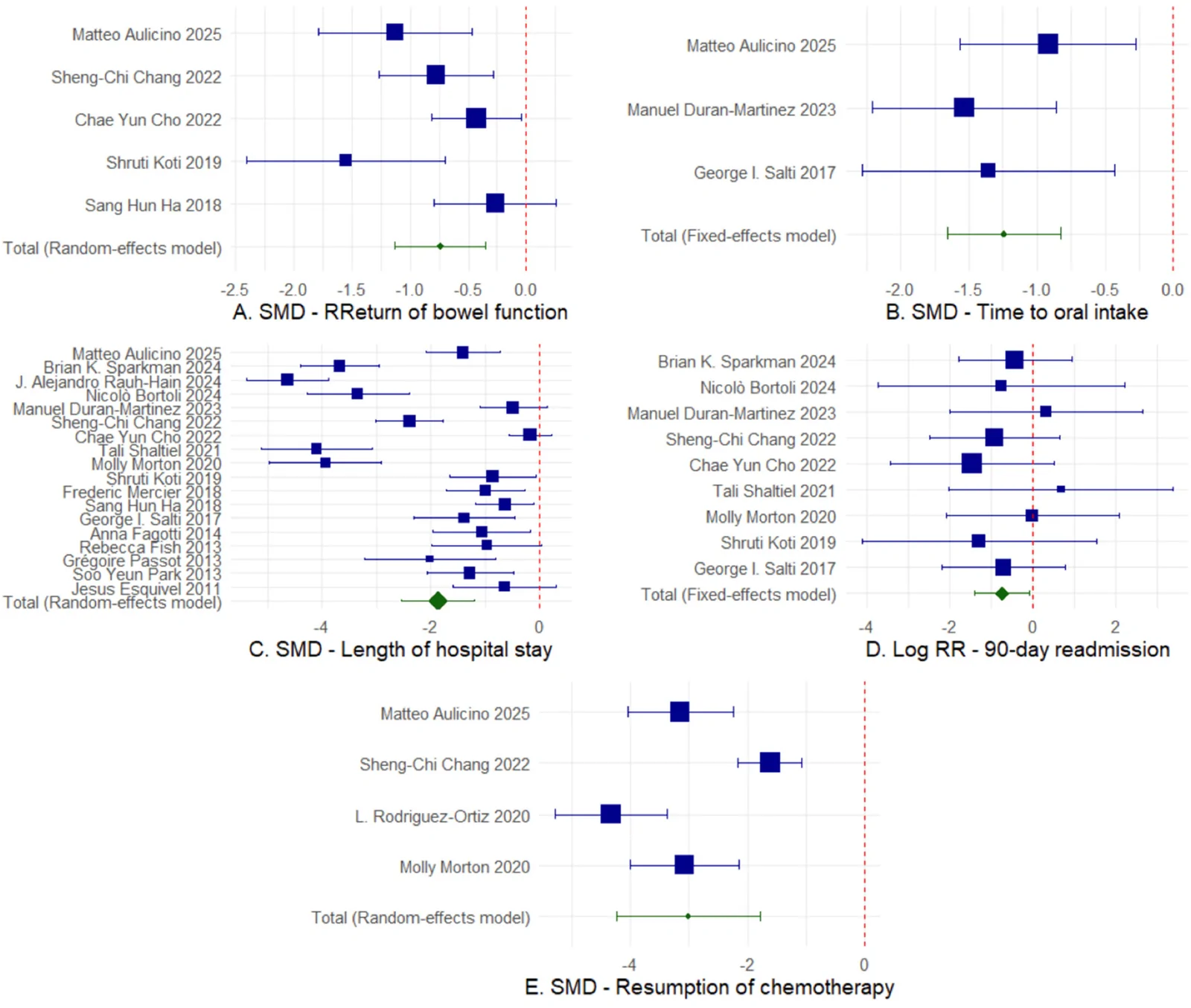
Forest plots comparing postoperative recovery outcomes between the M-CRS and O-CRS groups: (**A**) Return of bowel function; (**B**) Time to oral intake; (**C**) Length of hospital stay; (**D**) Ninety-day readmission; (**E**) Time to resumption of systemic chemotherapy. The red dashed line indicates no effect (null value = 0).

Postoperative recovery analysis between the M-CRS and O-CRS groups is shown in Figure 4.

### 3.4. Evaluation of Complications and Mortality

**Figure 5:**
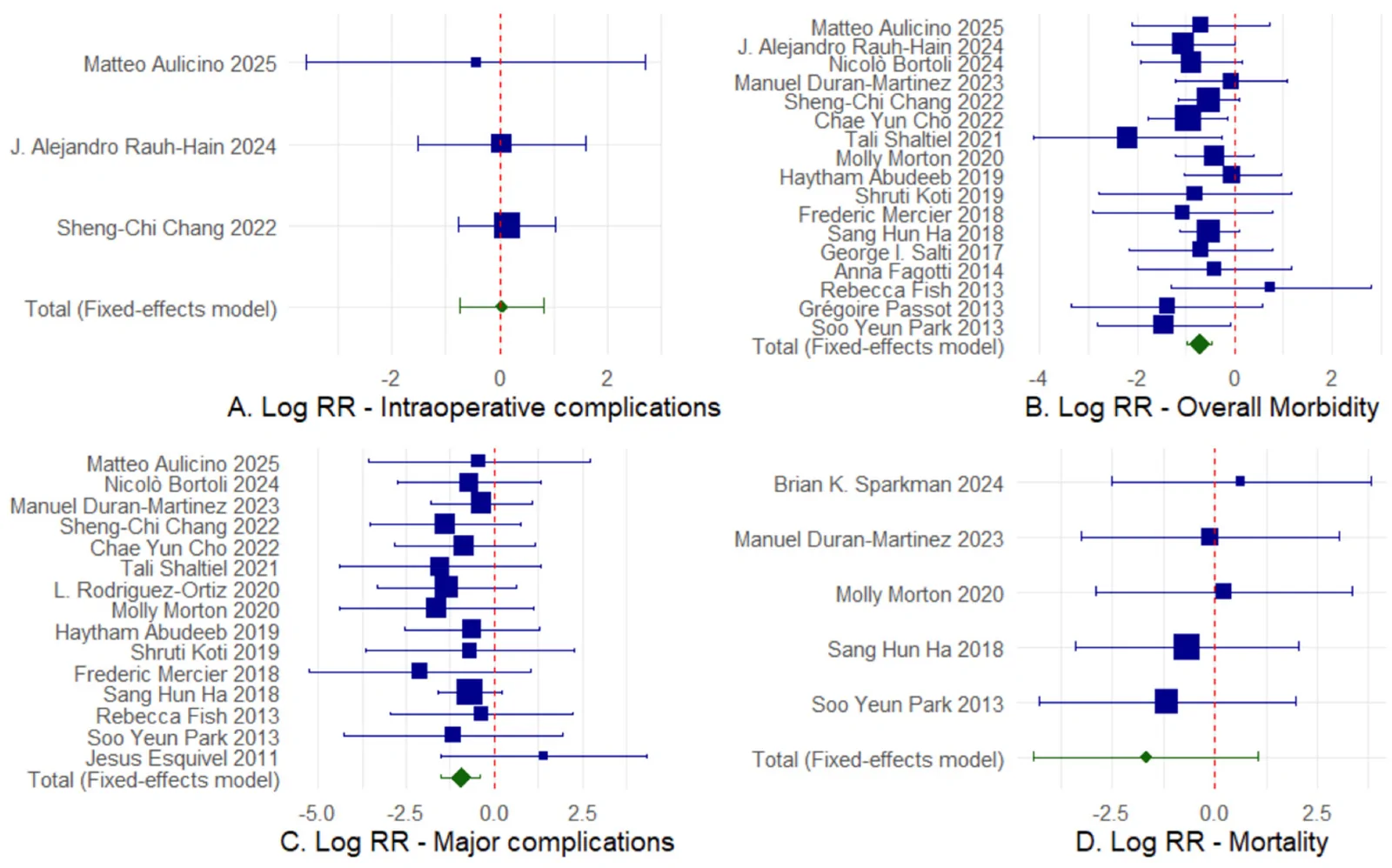
Forest plots comparing intraoperative and postoperative complications and mortality between the M-CRS and O-CRS groups: (**A**) Intraoperative complications; (**B**) Overall morbidity; (**C**) Major complications; (**D**) Mortality. The red dashed line indicates no effect (null value = 0).

Intraoperative complications were evaluated in 4 studies and did not differ significantly between the M-CRS and O-CRS groups (RR 1.043; 95% CI 0.482–2.258; *p* = 0.915). No heterogeneity was detected (*I*^2^ = 0%; *p* = 0.937), and no evidence of publication bias was observed (*p* = 0.792) (Figure 5A).

Overall morbidity, reported in 18 studies, is less frequent in the M-CRS group compared with the O-CRS group (RR 0.486; 95% CI 0.375–0.631; *p* < 0.001). No heterogeneity (*I*^2^ = 0%; *p* = 0.799) or publication bias (*p* = 0.403) was detected (Figure 5B).

Major complications were reported in 19 studies and occurred significantly less frequently in the M-CRS group (RR 0.549; 95% CI 0.223–0.675; *p* < 0.001). Heterogeneity was absent (*I*^2^ = 0%; *p* = 0.982), with no indication of publication bias (*p* = 0.721) (Figure 5C).

No statistically significant differences were observed for postoperative ileus (6 studies; RR 0.388; 95% CI 0.295–1.024; *p* = 0.059) or surgical site infection (4 studies; RR 0.203; 95% CI 0.032–1.289; *p* = 0.091).

Postoperative mortality was reported in 17 studies and did not differ significantly between groups (RR 0.186; 95% CI 0.012–2.842; *p* = 0.227). No significant heterogeneity was observed (*I*^2^ = 26.54%; *p* = 0.253), with no evidence of publication bias (*p* = 0.792) (Figure 5D).

### 3.5. Evaluation of Long-Term Outcomes

One-year disease-free survival (DFS) was reported in 7 studies and showed no statistically significant difference between the M-CRS and O-CRS groups (RR 1.054; 95% CI 0.943–1.234; *p* = 0.264). Heterogeneity was moderate (*I*^2^ = 68.10%; *p* = 0.008), and Egger’s regression test suggested potential publication bias (*p* = 0.078).

Three-year DFS, reported in 6 studies, did not differ significantly between groups (RR 0.967; 95% CI 0.661–1.388; *p* = 0.540). Heterogeneity was moderate (*I*^2^ = 56.25%; *p* = 0.044), with no evidence of publication bias (*p* = 0.754).

Five-year DFS was analyzed in 3 studies and similarly showed no statistically significant difference between the two groups (RR 1.336; 95% CI 0.842–2.120; *p* = 0.185). Heterogeneity was low (*I*^2^ = 13.88%; *p* = 0.313), and no publication bias was detected (*p* = 0.754).

**Figure 6:**
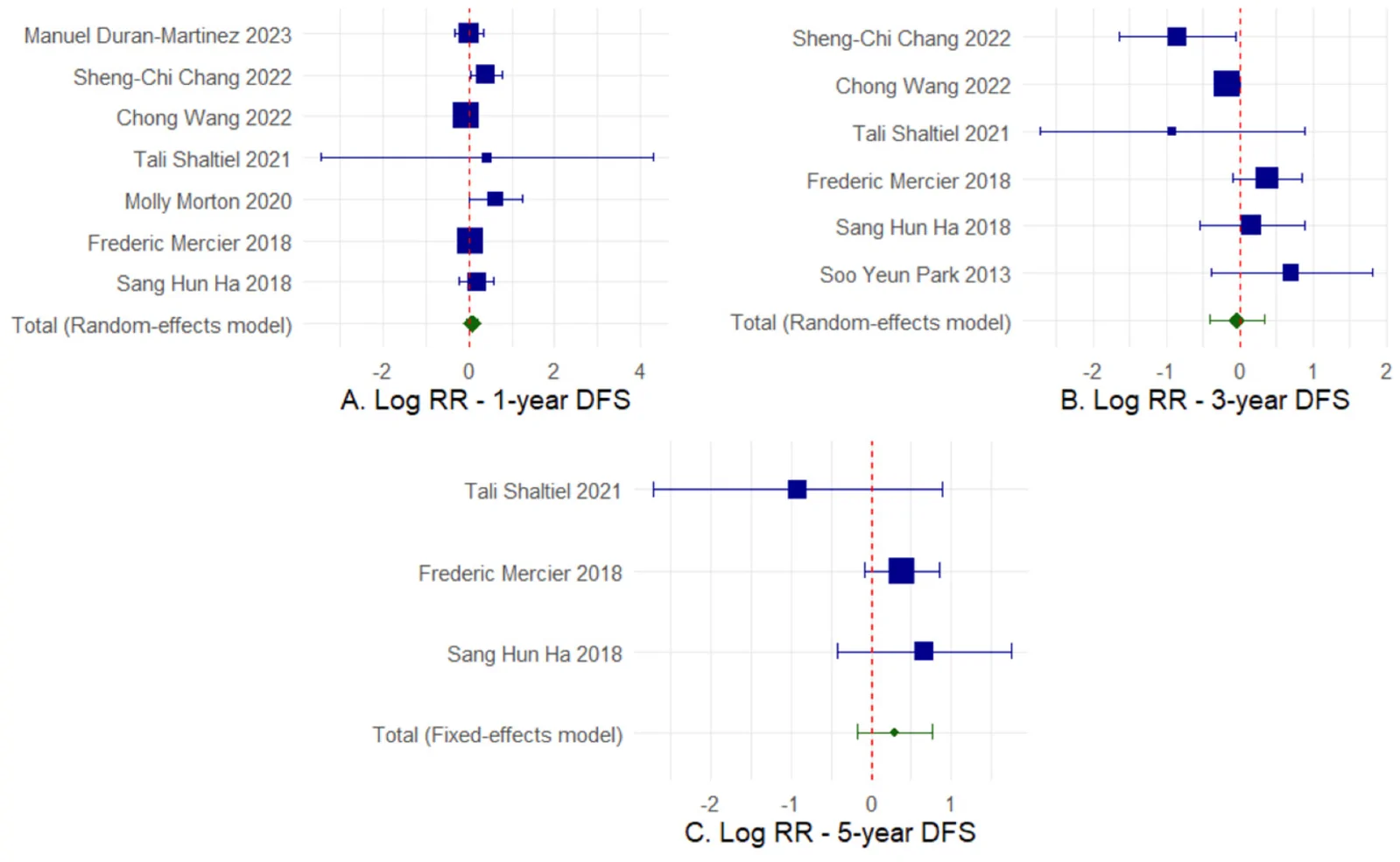
Forest plots comparing disease-free survival between the M-CRS and O-CRS groups: (**A**) 1-year DFS; (**B**) 3-year DFS; (**C**) 5-year DFS. The red dashed line indicates no effect (null value = 0).

Disease-free survival (DFS) comparisons between the M-CRS and O-CRS groups are illustrated in Figure 6.

One-year overall survival (OS) was reported in 5 studies and showed no statistically significant difference between the M-CRS and O-CRS groups (RR 1.103; 95% CI 0.992–1.119; *p* = 0.231). No heterogeneity was detected (*I*^2^ = 0%; *p* = 0.535), and Egger’s regression test did not suggest publication bias (*p* = 0.354).

Three-year OS, analyzed in 5 studies, did not differ significantly between groups (RR 1.090; 95% CI 0.938–1.267; *p* = 0.261). Heterogeneity was absent (*I*^2^ = 0%; *p* = 0.693), with no evidence of publication bias (*p* = 0.448).

**Figure 7:**
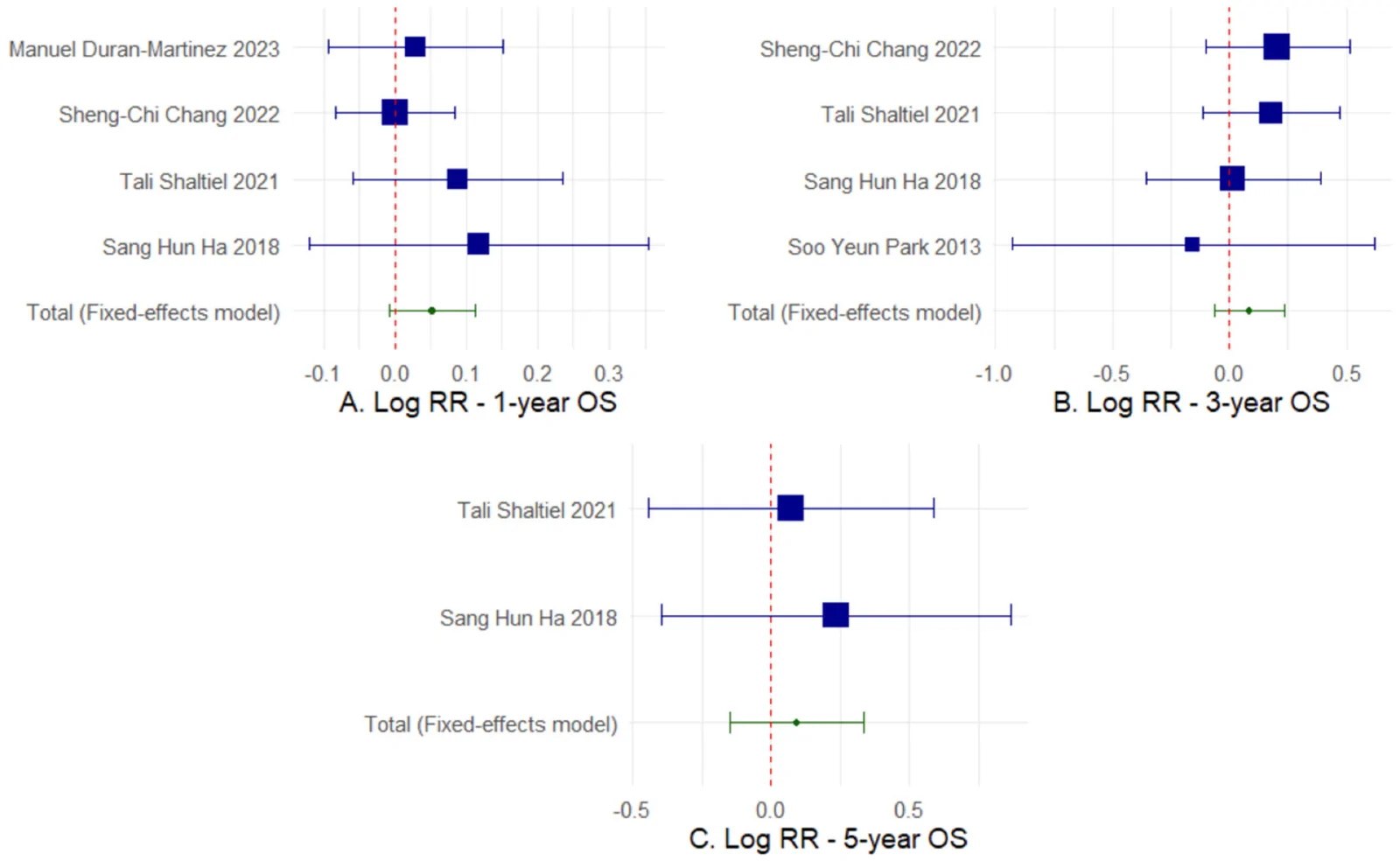
Forest plots comparing overall survival between the M-CRS and O-CRS groups: (**A**) 1-year OS; (**B**) 3-year OS; (**C**) 5-year OS. The red dashed line indicates no effect (null value = 0).

Five-year OS was reported in 3 studies and similarly showed no statistically significant differences between the M-CRS and O-CRS groups (RR 1.094; 95% CI 0.859–1.394; *p* = 0.465). No heterogeneity was detected (*I*^2^ = 0%; *p* = 0.651), and Egger’s regression test did not indicate publication bias (*p* = 0.694). Overall survival (OS) comparisons between the two groups are presented in Figure 7.

A comprehensive summary of surgical, perioperative, postoperative, and long-term oncologic outcomes comparing M-CRS and O-CRS, together with the corresponding GRADE certainty-of-evidence assessment for each outcome, is reported in Supplementary Material S3.

### 3.6. Disease Burden-Restricted Sensitivity Analysis

As differences in disease burden may have influenced the selection of the surgical approach and, consequently, the comparison between M-CRS and O-CRS, an additional sensitivity analysis focusing on PCI comparability between the two groups was performed. Of the 21 studies included in the meta-analysis, 8 were retrospective matched studies in which the M-CRS and O-CRS cohorts had comparable PCI values (studies no. 1, 4, 6, 8, 9, 13, 16, and 17). The analysis was therefore restricted to these studies, and all outcomes for which sufficient data were available for quantitative synthesis were re-evaluated.

Analysis of surgical procedures showed no statistically significant differences between M-CRS and O-CRS for colonic resection (5 studies; RR 0.835; 95% CI 0.598–1.166; *p* = 0.291; *I*^2^ = 18.54%), pelvic peritonectomy (3 studies; RR 1.028; 95% CI 0.764–1.381; *p* = 0.857; *I*^2^ = 46.53%), diaphragmatic peritonectomy (3 studies; RR 1.021; 95% CI 0.516–2.020; *p* = 0.952; *I*^2^ = 1.73%), hysterectomy/adnexectomy (4 studies; RR 1.026; 95% CI 0.449–2.347; *p* = 0.951; *I*^2^ = 76.98%), hepatic resection (3 studies; RR 1.154; 95% CI 0.506–2.632; *p* = 0.734; *I*^2^ = 0%), or stoma creation (4 studies; RR 0.658; 95% CI 0.367–1.179; *p* = 0.159; *I*^2^ = 0%), consistent with the primary analysis. In contrast, the significantly lower frequency of small-bowel resection observed with M-CRS in the primary analysis (RR 0.537; 95% CI 0.321–0.900; *p* = 0.018) lost statistical significance after restriction to matched studies with comparable PCI values (4 studies; RR 0.588; 95% CI 0.331–1.044; *p* = 0.070; *I*^2^ = 0%). Sensitivity analyses for gastric resection and splenectomy could not be performed because of insufficient data.

Among perioperative outcomes, operative time showed no statistically significant difference between M-CRS and O-CRS (7 studies; SMD −0.932; 95% CI −2.383 to 0.518; *p* = 0.208; *I*^2^ = 96.58%). The findings of the primary analysis were confirmed for intraoperative blood loss, which remained significantly lower in the M-CRS group (6 studies; SMD −2.723; 95% CI −3.793 to −1.653; *p* < 0.001; *I*^2^ = 90.33%), and for perioperative blood transfusions, which also remained significantly less frequent in the M-CRS group (4 studies; RR 0.590; 95% CI 0.384–0.909; *p* = 0.017; *I*^2^ = 0%). In contrast, the significantly higher CC0 rate observed in the M-CRS group in the primary analysis (RR 1.053; 95% CI 1.001–1.107; *p* = 0.045) lost statistical significance when the analysis was restricted to matched studies with comparable PCI values (4 studies; RR 1.026; 95% CI 0.933–1.129; *p* = 0.595; *I*^2^ = 0%).

Postoperative recovery outcomes largely retained the findings of the primary analysis. Return of bowel function remained significantly earlier in the M-CRS group (2 studies; SMD −0.902; 95% CI −1.297 to −0.506; *p* < 0.001; *I*^2^ = 0%), as did time to oral intake (2 studies; SMD −1.063; 95% CI −1.594 to −0.532; *p* < 0.001; *I*^2^ = 0%). Similarly, length of hospital stay remained significantly shorter in the M-CRS group (6 studies; SMD −2.255; 95% CI −3.128 to −1.382; *p* < 0.001; *I*^2^ = 85.13%), as did time to resumption of systemic chemotherapy (2 studies; SMD −2.339; 95% CI −3.832 to −0.846; *p* = 0.002; *I*^2^ = 87.63%). In contrast, the significantly lower 90-day readmission rate observed in the M-CRS group in the primary analysis (RR 0.482; 95% CI 0.249–0.935; *p* = 0.031) lost statistical significance when the analysis was restricted to matched studies with comparable PCI values (4 studies; RR 0.493; 95% CI 0.186–1.307; *p* = 0.155; *I*^2^ = 0%).

Analysis of complications confirmed the absence of statistically significant differences between M-CRS and O-CRS for intraoperative complications (2 studies; RR 1.043; 95% CI 0.429–2.536; *p* = 0.925; *I*^2^ = 0%) and postoperative ileus (3 studies; RR 0.333; 95% CI 0.093–1.191; *p* = 0.091; *I*^2^ = 0%). The findings of the primary analysis were also confirmed for overall morbidity, which remained significantly lower in the M-CRS group (7 studies; RR 0.430; 95% CI 0.274–0.675; *p* < 0.001; *I*^2^ = 0%), and for major complications, which also remained significantly less frequent in the M-CRS group (7 studies; RR 0.237; 95% CI 0.055–0.999; *p* = 0.050; *I*^2^ = 0%). Sensitivity analyses for surgical site infection and postoperative mortality could not be performed because of insufficient data.

Finally, long-term oncological outcomes showed no statistically significant differences between M-CRS and O-CRS in 1-year DFS (3 studies; RR 1.134; 95% CI 0.903–1.424; *p* = 0.281; *I*^2^ = 50.44%), 3-year DFS (3 studies; RR 0.670; 95% CI 0.421–1.065; *p* = 0.090; *I*^2^ = 26.21%), 1-year OS (2 studies; RR 1.041; 95% CI 0.973–1.114; *p* = 0.247; *I*^2^ = 23.70%), or 3-year OS (2 studies; RR 1.213; 95% CI 0.976–1.508; *p* = 0.081; *I*^2^ = 0%), confirming the findings of the primary analysis. Sensitivity analyses for 5-year DFS and OS could not be performed because of insufficient data.

The differences observed between the primary analysis and the disease burden-restricted sensitivity analysis are summarized in Table 2.

### 3.7. Tumor-Specific Sensitivity Analysis

Given the heterogeneity in primary tumor types across the included studies, a tumor-specific sensitivity analysis was conducted to assess long-term oncological outcomes. Of the 21 studies included in the meta-analysis, 10 exclusively enrolled patients with gastrointestinal malignancies, 4 exclusively enrolled patients with gynecological malignancies, and 7 included mixed primary tumor populations. Studies with mixed primary tumor populations were excluded from the tumor-specific analysis.

Among studies exclusively including gastrointestinal malignancies, no statistically significant differences between M-CRS/HIPEC and O-CRS/HIPEC were observed in 1-year DFS (4 studies; RR 1.158; 95% CI 0.900–1.490; *p* = 0.252; *I*^2^ = 27.15%), 3-year DFS (5 studies; RR 0.846; 95% CI 0.545–1.313; *p* = 0.456; *I*^2^ = 40.36%), or 5-year DFS (2 studies; RR 1.045; 95% CI 0.231–4.726; *p* = 0.955; *I*^2^ = 53.93%). Similarly, no statistically significant differences were observed in 1-year OS (3 studies; RR 1.030; 95% CI 0.962–1.105; *p* = 0.396; *I*^2^ = 0%), 3-year OS (4 studies; RR 1.140; 95% CI 0.953–1.365; *p* = 0.151; *I*^2^ = 0%), or 5-year OS (2 studies; RR 1.146; 95% CI 0.769–1.706; *p* = 0.505; *I*^2^ = 0%) (Supplementary Material S4).

Although four studies exclusively included patients with gynecological malignancies, a separate quantitative meta-analysis could not be performed because these studies did not provide sufficient long-term oncological outcome data at the prespecified time points.

### 3.8. Tumor Case-Mix-Restricted Sensitivity Analysis

To assess the potential influence of differences in primary tumor distribution between the M-CRS and O-CRS groups, a sensitivity analysis was performed including studies with adequately balanced primary tumor case-mix, defined as a maximum absolute standardized difference (Max |SMD|) < 0.10 across primary tumor categories or as both groups consisting exclusively of the same primary tumor type. Based on these criteria, 10 of the 21 included studies were selected for the sensitivity analysis. No statistically significant differences between M-CRS and O-CRS were observed for 1-year DFS (4 studies; RR 1.101; 95% CI 0.868–1.395; *p* = 0.429; *I*^2^ = 35.84%) or 3-year DFS (3 studies; RR 1.016; 95% CI 0.666–1.551; *p* = 0.941; *I*^2^ = 30.29%). Similarly, no statistically significant differences were observed for 1-year OS (2 studies; RR 1.049; 95% CI 0.941–1.170; *p* = 0.387; *I*^2^ = 0%) or 3-year OS (2 studies; RR 0.983; 95% CI 0.703–1.376; *p* = 0.921; *I*^2^ = 0%) (Supplementary Material S5). Sensitivity analyses for 5-year DFS and OS were not performed because only one eligible study was available for each of these endpoints.

## 4. Discussion

The role of minimally invasive approaches in cytoreductive surgery remains debated, with their use currently limited to highly selected patients treated in specialized, high-volume centers [48,49]. Despite encouraging perioperative outcomes, critical concerns remain regarding the ability of MIS to achieve complete cytoreduction and its impact on oncological outcomes over time [50]. Notably, the most recent meta-analysis, while providing a comprehensive assessment of perioperative outcomes, did not include a quantitative synthesis of long-term oncological endpoints [51].

To our knowledge, this is the first systematic review and meta-analysis providing a quantitative comparison between minimally invasive cytoreductive surgery combined with hyperthermic intraperitoneal chemotherapy (M-CRS/HIPEC) and the traditional open approach (O-CRS/HIPEC), integrating perioperative and postoperative recovery outcomes, long-term oncological outcomes, and the type and extent of surgical resections performed.

One of the key strengths of this meta-analysis is the procedure-level quantitative comparison of cytoreductive components between minimally invasive and open CRS/HIPEC. Rather than limiting the assessment to global perioperative or oncological endpoints, we systematically evaluated the main peritoneal and organ resections as a pragmatic surrogate of surgical complexity and technical reach, complementary to completeness of cytoreduction. This integrated, procedure-specific synthesis has been only partially explored in prior meta-analyses, which primarily focused on feasibility and short-term outcomes [51].

From a surgical standpoint, minimally invasive CRS was associated with a lower frequency of visceral and, particularly, small-bowel resections, whereas most other organ resections and peritonectomy procedures were comparable. Interestingly, the procedure less frequently performed in the minimally invasive setting is not inherently among the most technically demanding steps, which argues against an operative limitation. Overall, these patterns are more consistent with case-mix allocation and stricter selection than with intrinsic constraints of the minimally invasive platform, especially given that small-bowel and mesenteric involvement are key gatekeepers of CRS feasibility and radicality. This hypothesis is further supported by the finding that the lower frequency of small-bowel resection was no longer statistically significant when the analysis was restricted to matched studies with comparable PCI values. Accordingly, this finding should be interpreted as a marker of appropriate selection, whereby patients with mesenteric/small-bowel disease—generally unsuitable for a minimally invasive strategy—were appropriately excluded [52,53]. Within this high-expertise, selection-based framework, a minimally invasive approach appears able to deliver the main cytoreductive steps without systematic restrictions. Nonetheless, procedure reporting was heterogeneous across studies, and these findings should be viewed as resection-pattern signals rather than definitive equivalence of technical capability across all disease extents.

In the majority of the included studies, selection for a minimally invasive approach was based on a Peritoneal Cancer Index (PCI) below 10. However, experiences reported by Chang SC et al. [18], as well as by our research group in a recent matched retrospective study [42], did not apply a predefined PCI cut-off for patient selection. This observation entails a potential paradigm shift, whereby qualitative assessment of the anatomical distribution of peritoneal disease, such as multifocal mesenteric involvement or bulky lesions, may be more relevant than the numerical PCI score alone in guiding patient selection for M-CRS.

Within this context, the higher probability of achieving complete cytoreduction (CC0) observed in the M-CRS group should be interpreted with caution. Notably, the difference in CC0 rate was no longer statistically significant when the analysis was restricted to matched studies with comparable PCI values. Together with the corresponding loss of statistical significance for small-bowel resection, this finding suggests that the apparent differences in the extent and completeness of cytoreduction observed in the primary analysis may have been influenced, at least in part, by differences in baseline disease burden and case complexity. Accordingly, this finding does not indicate a lower intrinsic ability of open surgery to achieve oncological radicality. Rather, it likely reflects a differential allocation of case complexity, with more challenging patients preferentially undergoing open surgery, while those selected for M-CRS present with less extensive and more favorably distributed disease. Consequently, patient selection represents a potential source of selection bias and residual confounding, while simultaneously remaining an integral component of the clinical implementation of a minimally invasive strategy aimed at maximizing oncological radicality while preserving patient safety. Analysis of long-term oncological outcomes showed no statistically significant differences in OS or DFS between M-CRS and O-CRS at the evaluated time points. Importantly, this finding remained consistent across complementary sensitivity analyses designed to address two major sources of clinical heterogeneity: primary tumor biology and baseline peritoneal disease burden. In the tumor-specific analysis restricted to gastrointestinal malignancies, no statistically significant differences in DFS or OS were observed at 1, 3, or 5 years. A separate quantitative analysis of gynecological malignancies could not be performed because of insufficient reporting of long-term outcomes at the prespecified time points. Furthermore, when the analysis was restricted to studies with adequately balanced primary tumor case-mix between M-CRS and O-CRS, no statistically significant differences in DFS or OS were detected at 1 or 3 years. Similarly, the sensitivity analysis restricted to matched cohorts with comparable PCI values did not identify statistically significant differences in long-term survival outcomes.

Taken together, these complementary analyses suggest that the absence of statistically significant survival differences observed in the overall analysis was not exclusively attributable to heterogeneity in primary tumor distribution or numerical PCI imbalance between the two surgical groups. However, these findings should not be interpreted as evidence of oncological equivalence or non-inferiority, given the limited number of studies contributing to several survival endpoints, the predominantly retrospective nature of the available evidence, and the use of fixed-time survival estimates, which do not fully account for censoring or the time-to-event nature of these outcomes.

Nevertheless, when considered alongside the findings regarding completeness of cytoreduction, these results provide some reassurance regarding a longstanding concern surrounding M-CRS; namely, whether disease extent can be adequately assessed and complete cytoreduction achieved through a minimally invasive approach in appropriately selected patients. The observed oncological safety is likely driven by appropriate case selection and management within high-volume centers with experienced teams, supported by careful intraoperative decision-making (including timely conversion when needed) to maintain the likelihood of complete clearance. However, the loss of tactile feedback remains an inherent limitation of M-CRS. Intraoperative optical imaging techniques, such as fluorescence-guided navigation and photodynamic diagnosis, may improve the detection of peritoneal lesions and partially compensate for the absence of manual palpation; however, the available evidence remains limited and heterogeneous and requires further prospective validation [54,55]. Taken together, these considerations reinforce the need for a highly selective and expertise-driven implementation of minimally invasive CRS. In this context, laparoscopic cytoreductive surgery appears feasible and oncologically acceptable for selected patients when performed in highly specialized institutions with consolidated expertise in peritoneal surface malignancies and advanced laparoscopy, where skills from other complex minimally invasive oncologic procedures can be translated to CRS [56,57]. Nonetheless, technical feasibility should not be conflated with oncological indication, and open surgery should remain the reference approach for patients with extensive or anatomically complex peritoneal disease (e.g., high disease burden, mesenteric/small-bowel involvement, or anticipated extensive multivisceral resections).

Perioperative and postoperative recovery outcomes consistently favored M-CRS, in line with the findings of the previous meta-analysis by Pawar et al. [51]. The minimally invasive approach was associated with lower intraoperative blood loss and reduced transfusion requirements; moreover, the well-established recovery advantages of laparoscopy were maintained in the CRS setting, with earlier return of bowel function, shorter time to oral intake, and reduced length of hospital stay. Importantly, these findings remained statistically significant in the disease burden-restricted sensitivity analysis, together with earlier resumption of systemic chemotherapy, suggesting that the main perioperative and postoperative recovery advantages observed with M-CRS were not solely attributable to differences in disease burden between treatment groups. Although substantial heterogeneity was observed for some endpoints, the consistency in the direction of effect across the primary and restricted analyses, together with the concordance between the matched analysis and the only available RCT [47] for all directly comparable outcomes—including complete cytoreduction, intraoperative blood loss, length of hospital stay, intraoperative complications, and overall postoperative morbidity—supports the overall robustness of these findings. Notably, earlier resumption of systemic chemotherapy—originally evaluated in the present meta-analysis—represents a clinically relevant advantage, particularly in tumors with aggressive biological behavior, where shortening the surgery-to-chemotherapy interval may contribute to improved long-term oncological outcomes [18,42,58]. Conversely, the lower 90-day readmission rate observed in the primary analysis was no longer statistically significant after restriction to matched studies with comparable PCI values and should therefore be interpreted more cautiously.

From a safety standpoint, M-CRS was associated with significantly lower overall postoperative morbidity and major complication rates in both the primary analysis and the disease burden-restricted sensitivity analysis, while no significant difference in intraoperative complications was observed in either analysis. Postoperative mortality did not differ significantly between M-CRS and O-CRS in the primary analysis, although this outcome could not be reassessed in the disease burden-restricted analysis because of insufficient data. Overall, these findings suggest that the minimally invasive approach may reduce the surgical burden of CRS/HIPEC without increasing procedural risk, even after restricting the analysis to more comparable patient cohorts.

These findings should be interpreted in light of several limitations. The predominantly retrospective design of the included studies entails an inherent risk of selection bias, with minimally invasive approaches more frequently applied to patients with lower surgical complexity. The sensitivity analysis restricted to retrospective studies with matched populations allowed this potential bias to be partially mitigated. However, the persistence of substantial heterogeneity across several outcomes even in this analysis suggests the presence of additional sources of clinical and methodological variability, including patient characteristics, anatomical distribution and biology of the disease, surgical complexity, intraperitoneal treatment regimens, and center-specific patient selection practices. However, the ability to investigate the contribution of these factors through meta-regression was limited by the inconsistent reporting of potential moderators, which considerably reduced the number of studies available and precluded the reliable identification and quantification of the specific sources of the observed heterogeneity. Moreover, the “minimally invasive” group pooled heterogeneous techniques (laparoscopic/robotic/hand-assisted) and regimens (HIPEC vs. EPIC), potentially limiting the generalizability of pooled estimates, while robust subgroup analyses were constrained by study numbers and reporting. Another limitation is the heterogeneous distribution of primary tumor types across studies. Although this issue was partially addressed through sensitivity analyses based on primary tumor type and tumor case-mix, the limited number of studies and insufficient reporting of oncological outcomes, particularly for gynecological malignancies, do not allow a potential influence of tumor biology on the pooled estimates to be completely excluded. Moreover, OS and DFS were synthesized using survival proportions at fixed time points rather than hazard ratios. Although this approach allowed the available survival data to be quantitatively synthesized, fixed-time RRs do not account for censoring or the full time-to-event distribution and therefore provide less comprehensive information than HR-based analyses. In addition, the limited number of studies reporting long-term endpoints warrants cautious interpretation given potential inconsistencies in follow-up and reporting.

Nevertheless, a key strength of this meta-analysis is the concurrent quantitative assessment of perioperative outcomes, long-term oncologic endpoints, and procedure-level resection patterns across minimally invasive and open approaches—an integration only partially explored in previous evidence syntheses. Furthermore, the use of complementary sensitivity analyses based on disease burden, primary tumor type, and tumor case-mix allowed the robustness of the findings to be assessed with respect to some of the major sources of clinical heterogeneity and potential selection bias. This perspective enhances the clinical interpretability of the findings and supports a selective, risk-stratified implementation of M-CRS/HIPEC in patients treated at experienced, high-volume centers.

## 5. Conclusions

This meta-analysis supports a selective, expertise-driven application of minimally invasive CRS/HIPEC as a complementary approach rather than a replacement for open surgery. In appropriately selected patients, it is associated with meaningful perioperative and postoperative recovery benefits, without significant differences in cytoreductive adequacy or long-term outcomes. This strategy may therefore contribute to a more personalized and risk-adapted management of peritoneal surface malignancies.

## Figures and Tables

**Table 1:** Characteristics of the studies included in the meta-analysis.

N	Author/ Years	Type of Study	Type of MIS	Total PTS	M-CRS PTS	O-CRS PTS	HIPEC EPIC	M-CRS PCI	O-CRS PCI	M-CRS Primary Tumor	O-CRS Primary Tumor	Max |SMD|*
1	Matteo Aulicino (2025) [42]	Retrosp. Matched	LPS	45	15	30	HIPEC	3 (Range 1–16)	3 (Range 1–14)	Appendiceal primary tumor 3 (20%) CRC AdenoCa 8 (53.3%) Gastric cancer 2 (13.3%) Other 2 (13.3%)	Appendiceal primary tumor 2 (6.7%) CRC AdenoCa 22 (73.3%) Gastric cancer 3 (10%) Other 3 (10%)	0.424
2	Brian K. Sparkman (2024) [41]	Retrosp. Unmatched	Robotic	111	16	95	HIPEC	10 (IQR 7–6)	12 (IQR 8–20)	Appendiceal primary tumor 8 (50%) CRC AdenoCa 3 (18.8%) Ovarian cancer 4 (25%) Other 1 (6.3%)	Appendiceal primary tumor 35 (36.8%) CRC AdenoCa 30 (31.6%) Ovarian cancer 12 (12.6%) Other 18 (18.9%)	0.390
3	J.Alejandro Rauh-Hain (2024) [47]	RCT	LPS Robotic	100	49	51	HIPEC	__	__	Ovarian cancer 49 (100%)	Ovarian cancer 51 (100%)	Perfectly balanced
4	Nicolò Bortoli (2024) [40]	Retrosp. Matched	LPS	45	13	32	HIPEC	3 (IQR 2–4.5)	4 (IQR 2–6)	Appendiceal LAMN 6 (46.2%) Appendiceal adenoCa 2 (15.4%) CRC AdenoCa 2 (15.4%) Ovarian cancer 2 (15.4%) Other 1 (7.7%)	Appendiceal LAMN 7 (21.9%) Appendiceal adenoCa 1 (3.1%) CRC AdenoCa 15 (46.9%) Ovarian cancer 6 (18.8%) Other 3 (9.3%)	0.723
5	Manuel Duran-Martinez (2023) [46]	Retrosp. Unmatched	LPS	53	14	39	HIPEC	4.1 (SD ± 1.2)	6.3 (SD ± 2.9)	Ovarian cancer 14 (100%)	Ovarian cancer 39 (100%)	Perfectly balanced
6	Sheng-Chi Chang (2022) [18]	Retrosp. Matched	LPS HAL	68	34	34	HIPEC	6 (Range 1–13)	5 (Range 2–13)	Appendiceal adenoCa 3 (8.8%) CRC adenoCa 31 (91%)	Appendiceal adenoCa 2 (5.9%) CRC adenoCa 32 (94%)	0.113
7	Chae Yun Cho (2022) [45]	Retrosp. Unmatched	LPS	191	31	160	HIPEC	3 (Range 0–10)	7 (Range 0–10)	Appendiceal LAMN 13 (42%) Appendiceal AdenoCa 11 (35.4%) CRC adenoCa 5 (16%) Other 2 (6.5%)	Appendiceal LAMN 41 (26%) Appendiceal AdenoCa 27 (16.8%) CRC adenoCa 58 (36%) Ovarian cancer 11 (7%) Mesothelioma 13 (8%) Other (6)	0.763
8	Chong Wang (2022) [28]	Retrosp. Matched	LPS	66	33	33	HIPEC	1 (Range 0–3)	2 (Range 0–4)	Appendiceal LAMN 33 (100%)	Appendiceal LAMN 33 (100%)	Perfectly balanced
9	Tali Shaltiel (2021) [38]	Retrosp. Matched	LPS	48	16	32	HIPEC	5 (IQR 2–7)	5 (IQR 3–7)	Appendiceal primary tumor 4 (25%) CRC AdenoCa 8 (50%) Mesothelioma 2 (12.5%) Other 2 (12.6%)	Appendiceal primary tumor 12 (25%) CRC AdenoCa 16 (50%) Mesothelioma 5 (15.6%) Other 3 (9.5%)	0.184
10	L.Rodriguez-Ortiz (2020) [44]	Retrosp. Unmatched	LPS	60	18	42	HIPEC	3 (IQR 2.75–4)	5 (IQR 3–7.25)	Appendiceal LAMN 2 (11.8%) CRC adenoCa 6 (35.3%) Mesothelioma 2 (11.8%) Ovarian cancer 6 (35.6%) Other 1 (5.9%)	Appendiceal LAMN 6 (35.3%) CRC adenoCa 13 (32%) Mesothelioma 2 (4.9%) Ovarian cancer 15 (36.6%) Other 5 (12.2%)	0.251
11	Molly Morton (2020) [35]	Retrosp. Unmatched	Robotic LPS HAL	50	10	40	HIPEC	__	__	Ovarian cancer 10 (100%)	Ovarian cancer 40 (100%)	Perfectly balanced
12	Haytham Abudeeb (2019) [39]	Retrosp. Unmatched	Lap	84	55	29	HIPEC	0(Range 0–2)	0(Range 0–1)	Appendiceal LAMN 55 (100%)	Appendiceal LAMN 29 (100%)	Perfectly balanced
13	Shruti Koti (2019) [32]	Retrosp. Matched	Robotic LPS	33	9	24	HIPEC	5.83 (SD ± 3.68)	5.56 (SD ± 5.08)	Appendiceal primary tumor 5 (55.6%) CRC AdenoCa 3 (33.3%) Other 1 (11.1%)	Appendiceal primary tumor 9 (37.5%) CRC AdenoCa 11 (46%) Other 4 (16.7%)	0.368
14	Frederic Mercier (2018) [33]	Retrosp. Unmatched	LPS	43	32	11	HIPEC	2.5 (Range 0.9)	7 (Range 1–15)	Appendiceal LAMN 20 (65.5%) Mesothelioma 12 (37.5%)	Appendiceal LAMN 6 (54.6%) Mesothelioma 3 (27.3%) Ovarian cancer 2 (18.2%)	0.667
15	Sang Hun Ha (2018) [30]	Retrosp. Unmatched	LPS	63	42	21	HIPEC/ EPIC	PCI > 5 23.8%	PCI > 5 47.6%	CRC adenoCa 42 (100%)	CRC adenoCa 21 (100%)	Perfectly balanced
16	George I. Salti (2017) [37]	Retrosp. Matched	LPS HAL	22	11	11	HIPEC	4.1 (SD ± 5.1)	4.1 (SD ± 5.1)	Appendiceal LAMN 6 (54.6) Appendiceal adenoCa 2 (18.2) CRC adenoCa 3 (27.3%)	Appendiceal LAMN 4 (36.4%) Appendiceal AdenoCa 4 (36.4%) CRC adenoCa 3 (27.3%)	0.417
17	Anna Fagotti (2014) [43]	Retrosp. Matched	Robotic LPS	22	11	11	HIPEC	2	2	Ovarian cancer 11 (100%)	Ovary cancer 11 (100%)	Perfectly balanced
18	Rebecca Fish (2013) [31]	Retrosp. Unmatched	LPS HAL	17	10	7	HIPEC	__	__	Appendiceal LAMN 10 (100%)	Appendiceal LAMN 7 (100%)	Perfectly balanced
19	Grégoire Passot (2013) [34]	Retrosp. Unmatched	LPS	16	8	8	HIPEC	2.5 (Range 1–7)	8 (Range 4–13)	Appendiceal LAMN 5 (62.5%) Other 3 (37.5%)	Appendiceal LAMN 5 (62.5%) Other 3 (37.5%)	Perfectly balanced
20	Soo Yeun Park (2013) [29]	Retrosp. Unmatched	LPS	29	15	14	EPIC	__	__	CRC adenoCa 15 (100%)	CRC adenoCa 14 (100%)	Perfectly balanced
21	Jesus Esquivel (2011) [36]	Retrosp. Unmatched	LPS	18	10	8	HIPEC	4 (SD ± 3)	5 (SD ± 5)	Appendiceal LAMN 8 (80%) Mesothelioma 1 (10%) Other 1 (10%)	Appendiceal LAMN 5 (62.5%) CRC adenoCa 1 (12.5%) Mesothelioma 1 (12%) Other 1 (12.5%)	0.535

LPS Laparoscopic Surgery. HAL Hand-Assisted Laparoscopy. HIPEC Hyperthermic Intraperitoneal Chemotherapy. EPIC Early Postoperative Intraperitoneal Chemotherapy. LAMN Low-grade Appendiceal Mucinous Neoplasm. HCC Hepatocellular Carcinoma. CRC Colorectal Cancer. PCI Peritoneal Cancer Index. IQR interquartile range. SD standard deviation. RCT Randomized Controlled Trial. Retrospective matched: retrospective studies in which MIS and O cohorts were matched for baseline characteristics, including PCI. Max |SMD|*, maximum absolute standardized difference in primary tumor distribution between MIS and O groups.

**Table 2:** Outcomes showing a change in statistical significance in the disease burden-restricted sensitivity analysis.

Outcome	Primary Analysis	Disease Burden-Restricted Sensitivity Analysis	Change
Small-bowel resection	RR 0.537 (95% CI 0.321–0.900); *p* = 0.018	RR 0.588 (95% CI 0.331–1.044); *p* = 0.070	Significant → Non-significant
Complete cytoreduction (CC0)	RR 1.053 (95% CI 1.001–1.107); *p* = 0.045	RR 1.026 (95% CI 0.933–1.129); *p* = 0.595	Significant → Non-significant
90-day readmission	RR 0.482 (95% CI 0.249–0.935); *p* = 0.031	RR 0.493 (95% CI 0.186–1.307); *p* = 0.155	Significant → Non-significant

CC0, complete cytoreduction; CI, confidence interval; PCI, Peritoneal Cancer Index; RR, relative risk.

## Data Availability

All data analyzed in this systematic review and meta-analysis were obtained from previously published studies cited in the manuscript. The extracted dataset and the data generated during the analyses are available from the corresponding author upon reasonable request.

## Supplementary Materials

The following supporting information can be downloaded at: https://www.mdpi.com/article/10.3390/cancers18203310/s1, Supplementary Material S1: Complete search strategies for all databases; Supplementary Material S2: Risk-of-bias assessment of the included studies; Supplementary Material S3: GRADE assessment of certainty of evidence; Supplementary Material S4: Forest plots of DFS and OS in the primary tumor-specific sensitivity analysis; Supplementary Material S5: Forest plots of DFS and OS in the primary tumor case-mix-restricted sensitivity analysis.
