# Supplementary material for: Minimally Invasive Versus Open CRS/HIPEC for Peritoneal Surface Malignancies: Postoperative Recovery and Long-Term Outcomes—A Systematic Review and Meta-Analysis
Source: Cancers (Basel). 2026 Oct 14;18(20):3310. doi: 10.3390/cancers18203310 (PMC13702463; doi:10.3390/cancers18203310)

Supplementary Material S1: Complete Search Strategies for All Databases

PubMed

("cytoreduction"[Title/Abstract] OR "cytoreductive surgery"[Title/Abstract]) AND ("laparoscopy"[Title/Abstract] OR "laparoscopic"[Title/Abstract] OR "laparotomy"[Title/Abstract] OR "minimally invasive surgery"[Title/Abstract] OR "minimally invasive"[Title/Abstract] OR "robotic surgery"[Title/Abstract] OR "robot-assisted"[Title/Abstract] OR "robotic-assisted"[Title/Abstract]) AND ("HIPEC"[Title/Abstract] OR "hyperthermic intraperitoneal chemotherapy"[Title/Abstract]) AND ("neoplasm"[MeSH Terms] OR "cancer"[Title/Abstract] OR "tumor"[Title/Abstract] OR "carcinoma"[Title/Abstract])

Embase

('cytoreduction':ab,ti OR 'cytoreductive surgery':ab,ti) AND ('laparoscopy':ab,ti OR 'laparoscopic':ab,ti OR 'laparotomy':ab,ti OR 'minimally invasive surgery':ab,ti OR 'minimally invasive':ab,ti OR 'robotic surgery':ab,ti OR 'robot-assisted':ab,ti OR 'robotic-assisted':ab,ti) AND ('hipec':ab,ti OR 'hyperthermic intraperitoneal chemotherapy':ab,ti) AND ('neoplasm'/exp OR 'cancer':ab,ti OR 'tumor':ab,ti OR 'carcinoma':ab,ti)

Scopus

(TITLE-ABS-KEY("cytoreduction") OR TITLE-ABS-KEY("cytoreductive surgery")) AND (TITLE-ABS-KEY("laparoscopy") OR TITLE-ABS-KEY("laparoscopic") OR TITLE-ABS-KEY("laparotomy") OR TITLE-ABS-KEY("minimally invasive surgery") OR TITLE-ABS-KEY("minimally invasive") OR TITLE-ABS-KEY("robotic surgery") OR TITLE-ABS-KEY("robot-assisted") OR TITLE-ABS-KEY("robotic-assisted")) AND (TITLE-ABS-KEY("HIPEC") OR TITLE-ABS-KEY("hyperthermic intraperitoneal chemotherapy")) AND (TITLE-ABS-KEY("cancer") OR TITLE-ABS-KEY("tumor") OR TITLE-ABS-KEY("carcinoma") OR TITLE-ABS-KEY("neoplasm"))

Web of science

TS=("cytoreduction" OR "cytoreductive surgery") AND TS=("laparoscopy" OR "laparoscopic" OR "laparotomy" OR "minimally invasive surgery" OR "minimally invasive" OR "robotic surgery" OR "robot-assisted" OR "robotic-assisted") AND TS=("HIPEC" OR "hyperthermic intraperitoneal chemotherapy") AND TS=("cancer" OR "tumor" OR "carcinoma" OR "neoplasm")

Cochrane Library

(cytoreduction OR "cytoreductive surgery") AND (laparoscopy OR laparoscopic OR laparotomy OR "minimally invasive" OR "robotic surgery" OR "robot-assisted") AND (HIPEC OR "hyperthermic intraperitoneal chemotherapy") AND (cancer OR neoplasm OR tumor OR carcinoma)

Supplementary Material S2

Table S1. Risk-of-bias assessment of retrospective cohort studies using the Newcastle-Ottawa Scale.

Author, Years		Selection				Comparability		Outcomes			Final Score
		1	2	3	4	1	2	1	2	3	
Matteo Aulicino	2025	*	*	*	*	*	*	*	-	*	8
Brian K. Sparkman	2024	*	*	*	*	*	-	*	-	*	7
Nicolò Bortoli	2024	*	*	*	*	*	*	*	-	*	8
Manuel Duran-Martinez	2023	*	*	*	*	*	-	*	*	*	8
Sheng-Chi Chang	2022	*	*	*	*	*	*	*	*	*	9
Chae Yun Cho	2022	*	*	*	*	*	-	*	-	*	7
Chong Wang	2022	*	*	*	*	*	*	*	*	*	9
Tali Shaltiel	2021	*	*	*	*	*	-	*	*	*	8
L.Rodriguez-Ortiz	2020	*	*	*	*	*	-	*	*	*	8
Molly Morton	2020	*	*	*	*	*	-	*	*	*	8
Haytham Abudeeb	2019	*	*	*	*	*	-	*	-	*	7
Shruti Koti	2019	*	*	*	*	*	-	*	-	*	7
Frederic Mercier	2018	*	-	-	*	*	-	*	*	*	6
Sang Hun Ha	2018	*	*	*	*	*	-	*	*	*	8
George I. Salti	2017	*	*	*	*	*	-	*	-	*	7
Anna Fagotti	2014	*	*	*	*	*	-	*	-	*	7
Rebecca Fish	2013	*	*	*	*	*	-	*	-	*	7
Grégoire Passot	2013	*	*	*	*	*	-	*	-	*	7
Soo Yeun Park	2013	*	*	*	*	*	-	*	*	*	8
Jesus Esquivel	2011	*	-	-	*	*	-	*	*	*	6

Table S2. Risk-of-bias assessment of the randomized clinical trial using RoB 2.0.

Author, Years		D1	D2	D3	D4	D5	Overall
Rauh-Hain	2024	Low	Some concerns	Low	Low	Low	Some concerns

Supplementary Material S3: GRADE Assessment of Certainty of Evidence for Surgical, Perioperative, Postoperative, and Long-Term Outcomes Comparing Minimally Invasive and Open CRS/HIPEC

Table S3. GRADE assessment of certainty of evidence for the outcomes comparing minimally invasive and open CRS/HIPEC.

Outcome	No. of Studies	Effect Measure (95% CI)	P Value	Result (M-CRS vs O-CRS)	I^2	Certainty	Reason for rating
**Surgical procedures**							
Peritoneal resections	5	SMD –0.199 (–1.300 - 0.901)	0.722	Not significant	93.4%	Very low	Serious inconsistency across studies and imprecision
Small bowel resections	8	RR 0.537 (0.321–0.900)	0.018	↓ M-CRS	0%	Low	Possible selection bias
**Perioperative outcomes**							
Operative time	18	SMD 0.195 (–0.692 to 1.082)	0.667	Not significant	97.3%	Very low	Serious inconsistency across studies and indirectness
Complete cytoreduction (CC0)	13	RR 1.053 (1.001–1.107)	0.045	↑ M-CRS	0%	Low	Possible selection bias
Intraoperative blood loss	14	SMD –1.838 (–2.501 to –1.175)	<0.001	↓ M-CRS	91%	Low	Serious inconsistency across studies
Perioperative blood transfusions	9	RR 0.588 (0.417–0.829)	0.002	↓ M-CRS	0%	Moderate	Large effect size with no serious inconsistency
**Postoperative recovery**							
Return of bowel function	5	SMD –0.740 (–1.128 to –0.351)	<0.001	Earlier in M-CRS	46.9%	Low	Imprecision due to few studies
Time to oral intake	3	SMD –1.242 (–1.660 to –0.824)	<0.001	Earlier in M-CRS	0%	Low	Imprecision due to few studies
Length of hospital stay	20	SMD -1.867 (-2.532 to - 1.201)	<0.001	Shorter in M-CRS	91.2%	Low	Serious inconsistency across studies
90-day readmission	9	RR 0.482 (0.249–0.935)	0.031	↓ M-CRS	0%	Moderate	Large effect size with no serious inconsistency
Time to chemotherapy resumption	5	SMD –2.479 (–3.801 to –1.156)	<0.001	Earlier in M-CRS	92.1%	Low	Inconsistency across studies
**Complications /mortality**							
Intraoperative complications	4	RR 1.043 (0.482–2.258)	0.915	Not significant	0%	Low	Imprecision due to few studies
Overall morbidity	18	RR 0.486 (0.375–0.631)	<0.001	↓ M-CRS	0%	Moderate	Large effect size with no serious inconsistency

Major complications	19	RR 0.549 (0.223–0.675)	<0.001	↓ M-CRS	0%	Moderate	Large effect size with no serious inconsistency
Postoperative mortality	17	RR 0.186 (0.012–2.842)	0.227	Not significant	26.5%	Low	Imprecision due to few events
**Long-term outcomes**							
1-year DFS	7	RR 1.079 (0.943–1.234)	0.264	Not significant	68.1%	Low	Imprecision due to few studies
3-year DFS	6	RR 0.967 (0.661–1.388)	0.540	Not significant	56.2%	Low	Imprecision due to few studies
5-year DFS	3	RR 1.336 (0.842–2.120)	0.185	Not significant	13.8%	Low	Imprecision due to few studies
1-year OS	5	RR 1.054 (0.992–1.119)	0.231	Not significant	0%	Low	Imprecision due to few studies
3-year OS	5	RR 1.090 (0.938–1.267)	0.261	Not significant	0%	Low	Imprecision due to few studies
5-year OS	3	RR 1.094 (0.859–1.394)	0.465	Not significant	0%	Low	Imprecision due to few studies

DFS, disease-free survival; OS, overall survival; RR, relative risk; SMD, standardized mean difference; I^2, heterogeneity.

Supplementary Material 4. Forest plots of the primary tumor-specific sensitivity analysis comparing disease-free survival (DFS) and overall survival (OS) between the M-CRS and O-CRS groups: (A) 1-year DFS; (B) 3-year DFS; (C) 5-year DFS; (D) 1-year OS; (E) 3-year OS; (F) 5-year OS. The red dashed line represents the line of no effect (null value = 0).

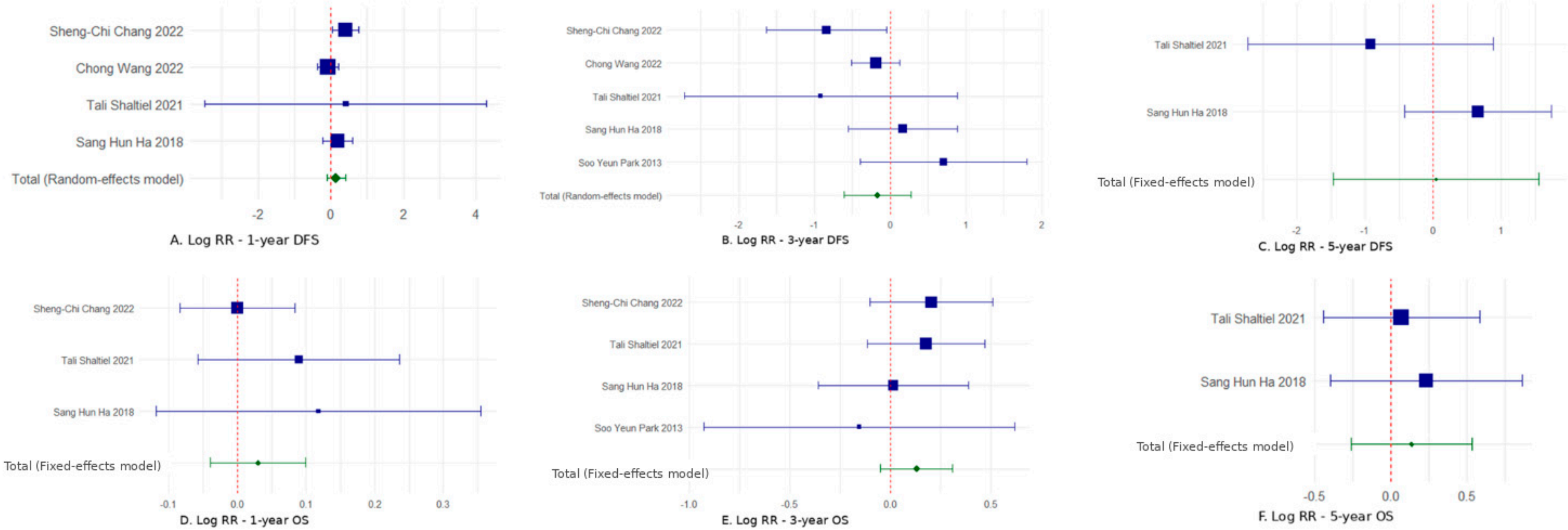

Supplementary Material 5. Forest plots of the primary tumor case-mix–restricted sensitivity analysis comparing disease-free survival (DFS) and overall survival (OS) between the M-CRS and O-CRS groups: (A) 1-year DFS; (B) 3-year DFS; (C) 1-year OS; (D) 3-year OS. The red dashed line represents the line of no effect (null value = 0).

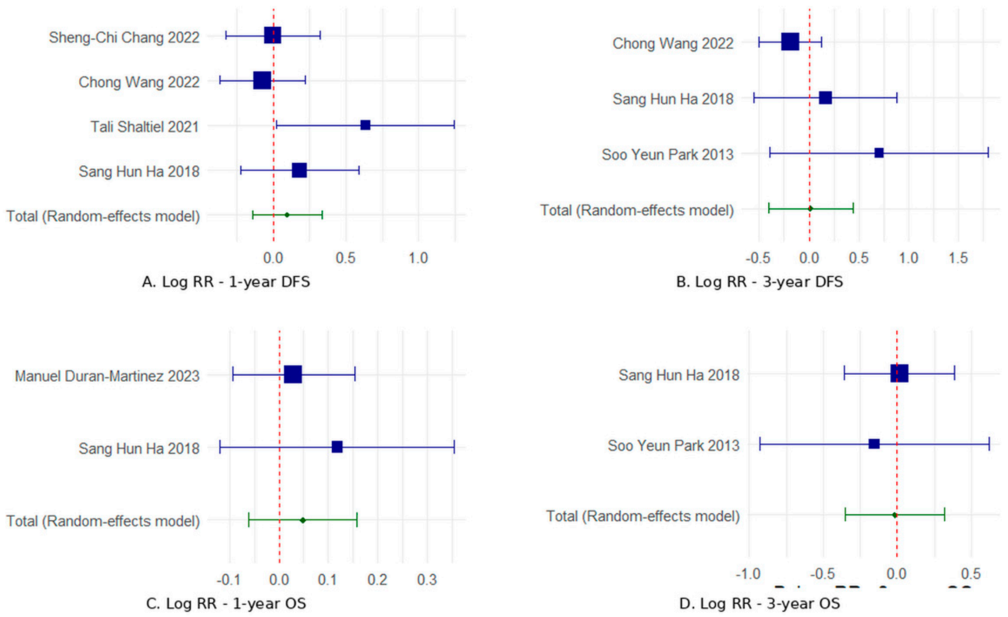

Supplement: Supplementary file 1 [file cancers-18-03310-s001.zip › cancers-4562490-supplementary.pdf]
